# Multilayer Smart Holographic Label with Integrated RFID for Product Security and Monitoring

**DOI:** 10.3390/mi14030692

**Published:** 2023-03-21

**Authors:** Cătălin Pârvulescu, Veronica Anăstăsoaie, Roxana Tomescu, Martino Aldrigo, Dana Cristea

**Affiliations:** National Institute of Research and Development in Microtechnology—IMT Bucharest, 077190 Voluntari, Romania

**Keywords:** holography, holographic label, RFID antenna, security label, UHF RFID, multilayer smart security labels

## Abstract

Counterfeiting presents a major economic problem and an important risk for the public health and safety of individuals and countries. To make the counterfeiting process more difficult, and to ensure efficient authentication, a solution would be to attach anti-counterfeit labels that include a radio frequency identification (RFID) element to the products. This can allow real-time quality check along the entire supply chain. In this paper we present the technology optimized to obtain a multilayer holographic label with a high degree of security, patterned on a thin zinc sulfide film of a semi-transparent holographic foil rather than on the standard substrate for diffractive optical elements (metallized foil). The label is applied onto the product surface or packaging for anti-counterfeit protection. The developed multilayer structure contains various elements such as: a holographic background, nanotext-type elements, holographic elements, and an RFID antenna. The employed semi-transparent holographic foil offers the RFID antenna the possibility to transmit the electromagnetic signal through the label and thus to maximize the antenna footprint, achieving up to 10 m reading distance, with a 6 cm × 6 cm label, much smaller than the commercial standard (minimum 10 cm × 10 cm).

## 1. Introduction

Illicit drug trafficking and trade in counterfeit goods occupy the first two places in terms of revenue at the global level, the latter adding up to a value of 2.2 trillion dollars annually [1]. These goods are spread in most markets such as the pharmaceutical market, clothing, automotive components, microelectronic components, and fuel. By increasing the level of counterfeiting, the industry sector records high financial losses, leading to an annual loss of tens of thousands of jobs. Moreover, widespread counterfeiting among products, from consumer goods to high-end technologies, has negative effects on the health and safety of the population. For example, counterfeit microelectronic components can cause satellites crash or military systems malfunction causing major security issues [2,3,4].

Usually, the protection against counterfeiting is carried out by attaching security labels to the products which include holographic elements that produce various visual effects, filigree (watermark), barcodes, QR codes, micro/nano-text, or labels printed with special inks (colorimetric, fluorescent, based on luminescent quantum dots) [5,6,7,8,9,10,11]. The anti-counterfeiting technologies used must be difficult to duplicate, yet easy to implement, and be visually identifiable and detectable if tampered with [12]. These technologies can be divided into direct technologies, which are clearly visible to the consumer, or indirect technologies, which are not visible to the naked eye. Direct technology enables end users to visually verify the originality of the packaging and consists of holograms, watermarks, barcodes, RFID, and tamper-evident seals. Covert technologies include special inks/chemical or mechanical methods [12,13]. Examples of covert technologies are invisible digital watermarks and microtext. This text has dimensions at the micro- and nanoscale and can be inserted at specific coordinates on the holographic background of the label. These elements present a high security level, being extremely difficult to identify and to replicate [14]. Usually, holographic tags are fabricated using only one of these technologies. 

Another efficient solution to these problems is to attach onto the product anti-counterfeiting labels that include a radio frequency identification (RFID) element that can protect and monitor the item.

On a large scale, the use of RFID systems has fully demonstrated its benefits, being used in transport, health, commerce, and industry to track, identify and control objects and services in a precise and reliable way [15]. RFID has gained wide interest because it represents a superior alternative to traditional bar codes, which are usually read using a laser-based optical scanner and are easy to reproduce. The advantage of RFID is that it is readable even when it is hidden behind a holographic security tag, sometimes from several meters. RFID has reached a level of technological maturity offering promising results at ultra-high frequencies (UHF) and in the microwave range, e.g., 868 MHz (Europe)/915 MHz (USA)/950 MHz (Japan), 2.45 and 5.8 GHz, with a maximum power between 5 mW and 4 W of so-called equivalent isotropically radiated power (EIRP).

The aim of this work was the development of an innovative technology to manufacture a multilayer holographic smart label, consisting of holographic elements patterned on a semi-transparent foil (holographic background, foreground, and nanotext-type security elements), and a specifically designed passive RFID structure. Furthermore, the resulting smart tag obtained integrates the two anti-counterfeiting technologies and contributes to product protection and surveillance throughout the chain, from producer to customer. The proposed RFID element is attached to the holographic label, thus increasing its degree of security and making the counterfeiting process almost impossible. Usually, when developing a security label with RFID elements, the diffractive optical elements (DOE) composing the holographic foreground and background are defined in a metalized polymer foil. In those cases, to allow the RFID to work properly, the antenna has to be positioned in a window opened in the metallic film [16] or on the exterior frame of the holographic layout, and thus increasing considerably the dimensions of the total security label [17]. In our case, the semi-transparent foil was selected to minimize the smart label area and at the same time to attain competitive reading distance. A second innovative aspect of this work is the fabrication of the designed RFID antenna on a Kapton HN thin foil.

Previously, we developed low-cost technologies for the manufacture of (a) holographic labels with a security key composed of alphanumeric symbols engraved at the exact coordinates and metallic microparticles with various geometries and alphanumeric elements, and (b) metallic microtags combined with other security elements such as a holographic background with various visual effects and small-dimension microtexts with alphanumeric elements. These technologies were developed for the fabrication of classical labels with DOE configured in a metalized polymer foil [18,19].

**Figure 1:**
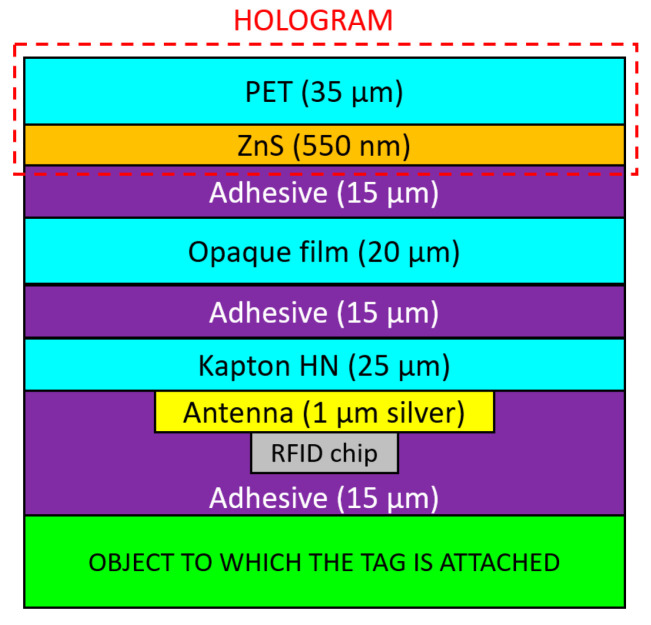
Cross-section of the proposed multilayer label.

Here we propose a multilayer security label with DOE patterned on a thin zinc sulfide film of a semi-transparent holographic foil integrated with a spiral shape RFID antenna. The previously developed technologies were optimized by adjusting the process parameters to the new materials and requirements of the current application. Figure 1 represents the cross-section of the proposed multilayer label illustrating the used materials and the thickness of each layer. This structure offers the possibility to transmit the electromagnetic signal through the label and thus to maximize the antenna footprint, allowing up to 10 m reading distance, with a 6 cm × 6 cm label, much smaller than the commercial standard (minimum 10 cm × 10 cm). The design of the RFID included in this multilayer structure considers several requirements such as: (i) optimization of performance/antenna size ratio, and (ii) minimization of the surrounding layers influence. The technology we propose for the fabrication of the smart multilayer holographic label ensures an optimum integration of all elements and can be easily adapted for mass production. To further increase the security level of the developed tag, the holographic foil is designed to degrade when prying apart from the product/package, making it impossible to reattach.

The prototype of the multilayer label was experimentally tested in a real-world indoor scenario to determine the maximum identification distance.

## 2. RFID Antenna: Design, Modelling, Simulation, and Fabrication

**Figure 2:**
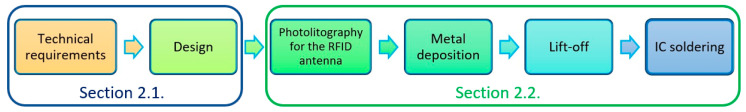
Main stages for the development of an RFID antenna.

The main stages for the development of the RFID antenna are: the establishing of technical requirements, designing the RFID antenna, photolithography, metal deposition, lift off, and soldering the integrated circuit (IC) RFID commercial chip. These steps are illustrated schematically in Figure 2.

### 2.1. Design, Modelling, and Simulation of RFID Elements

The RFID antenna was designed, modelled, and simulated using the three-dimensional electromagnetic simulator CST Microwave Studio (MathWorks, Natick, MA, USA) with the following technological characteristics and specifications: (i) reference band: 860–960 MHz; (ii) RFID tag type: passive (no integrated battery); (iii) materials used: adhesive, Polyethylene terephthalate (PET), zinc sulfide (ZnS); (iv) possibility to bend the label at an angle of no more than 90°; (v) initial reading distance: minimum 6 m; (vi) maximum 10 × 10 cm antenna footprint. Regarding the RFID chip, a commercial model NXP UCODE 8/8 m (Hitachi, NXP Semiconductor, Eindhoven, The Netherlands) was used, with a typical impedance at 915 MHz (in the case of fixing on the antenna’s large surface supports) of ZC = 19 − 234 j Ω.

When designing an RFID tag, the most significant challenges are maximizing the ratio performance/antenna size and minimizing of the influence of the surrounding layers. The design should also consider that the shape and the dielectric characteristics of material of the platforms on which it will be attached can significantly affect parameters such as input impedance, resonance frequency, and antenna gain [20].

The first step for a good design of an antenna is to define the characteristics of the materials used for the fabrication of RFID. Table 1 shows the values of the permittivity, ε, and the tangent of the loss angle, tan δ (or, equivalently, of the conductivity, σ) for these materials. It should be emphasized that the range of values for tan δ (which represents a quantifiable measure of dielectric losses in the respective material) in the case of polyethylene terephthalate (PET) includes two borderline situations, the best (1.4 × 10^−6^) and the worst (0.002).

The antenna was designed as a radiating element in free space, without other layers around it. It was taken into account that the target was to reduce the total dimensions below commercial standards (of approx. 10 cm × 10 cm); thus, a total area (occupied by the antenna surface) of only 6 cm × 6 cm was taken into account, and for the actual antenna, a logarithmic spiral type antenna was chosen, a choice made for the following reasons:

I. It is a symmetrical antenna (like the dipole or “bow-tie” antenna), which is the natural choice for an RFID application, with the respective chip fixed between the lines of the symmetrical power port.
II. It provides an extremely wide frequency band that depends on the smallest radius (for the highest frequency) and the largest radius (for the lowest frequency).
III. It has a high radiation efficiency, which is a great advantage considering the fact that the integration of the antenna between several layers (with reduced thickness compared to the wavelength in free space) of the dielectric/semiconductor type inevitably leads to a dramatic decrease in power actually radiated to the reader device.
IV. It radiates in a plane perpendicular to the antenna in both directions and equally.
V. Theoretically, the polarization of the radiated field is circular, which can be an advantage in certain applications wherein the receiving antenna must be able to receive a signal regardless of the orientation of the transmitting antenna.

For a logarithmic spiral antenna (or equiangular spiral or “spira mirabilis”), the following Equation (1) describes each arm (in polar coordinates) [21]:

(1) $$r=r_{0}e^{a\left(\varphi -\delta \right)}$$

where r_0_ is the initial radius of the spiral, a is the growth rate of the spiral, φ is the angular growth of the spiral, and δ is the phase shift term.

In our case, r_0_ = 1 mm, a = 0.1, φ = 1°, δ = 60°, while the total number of rotations is 5.2 and the metallization used is Ag (thickness of 1 μm).

In order to obtain an antenna that works at least from 860 MHz upwards in an area with maximum dimensions of 6 cm × 6 cm, some rectangular Ag tracks were added that surround the logarithmic spiral, thus allowing us to obtain an electrical length high enough to reach the lowest working frequency. This technique, intended to miniaturize the antenna, is a practice used quite often in the literature and commercial applications, with the aim of obtaining antennas conforming to the dimensions of the RFID tag.

**Figure 3:**
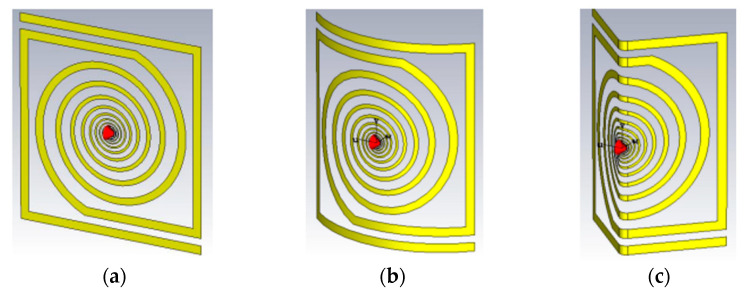
RFID antenna design in configuration: (**a**) planar, (**b**) curved at an angle of 90°, and (**c**) bent at an angle of 90°.

**Figure 4:**
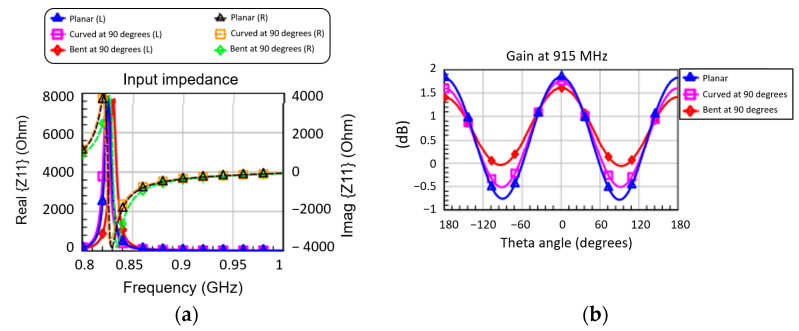
Electromagnetic simulations for (**a**) input impedance (left vertical axis: real part; right vertical axis: imaginary part) and (**b**) gain at 915 MHz, depending on the angle θ, for the three logarithmic spiral antenna configurations.

Figure 3a shows the logarithmic spiral antenna, as it was designed and simulated in CST Microwave Studio. Once the basic antenna was designed and simulated, its shape was modified either by curving at an angle of 90° (Figure 3b) or by bending at an angle of 90° (Figure 3c), with the aim of predicting the effect of curvature/bending on the performance of the spiral in terms of input impedance (Figure 4a) and gain (Figure 4b), two important parameters sensitive to bending.

From Figure 4a,b, it can be seen that the performance changes depending on whether the bends are negligible in the range of values of the angle θ (in the plane perpendicular to the antenna) between −60° and 60°. Therefore, we chose to simulate the entire multi-layer RFID antenna given in Figure 1 in a planar configuration (because the curvature/bending significantly prolongs the calculation time of the electromagnetic simulator). It should be emphasized that the antennas designed in free space already have a reactive input impedance with a small real part (resistive, Figure 4a, left vertical axis) and quite a large imaginary part (capacitive, Figure 4a, vertical axis right) in the entire band of interest. This aspect is important for the integration of the antenna in the multilayer chip structure. Furthermore, in Figure 4b the angle θ = 0° corresponds to the direction perpendicular to the hologram (Figure 1). The radiation efficiency in all three cases presented in Figure 3 has an approximately constant value of 78% at 915 MHz.

**Figure 5:**
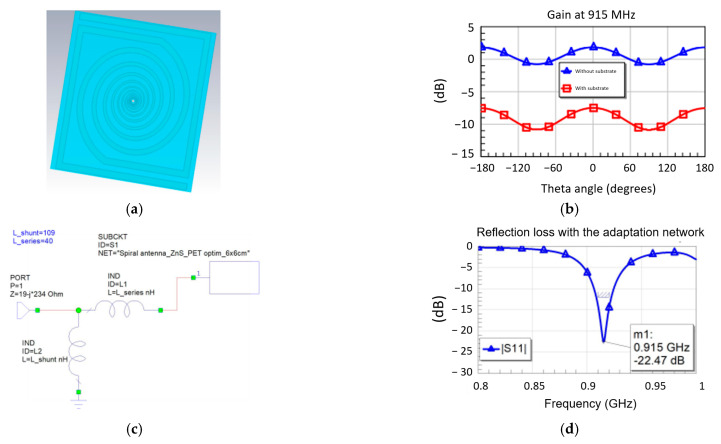
(**a**) The electromagnetic design of the RFID antenna integrated in the multilayer structure from Figure 1; (**b**) comparison between the gain at 915 MHz of the antenna in free space and the integrated antenna; (**c**) the adaptation circuit and (**d**) circuit simulations of the antenna integrated with the chip.

Figure 5a shows the antenna with the layout presented in Figure 3a and integrated in the multilayer structure given in Figure 1. In this case, due to the fact that the spiral is completely surrounded by dielectric/semiconductor-type materials, the efficiency of the radiated field η drops significantly, which leads to a decrease in the gain G (Figure 5b), despite keeping a good value for the directivity D (since G = η·D). However, the great advantage regarding input impedance must be emphasized: in this case, the antenna has a real (resistive) part of approx. 42 Ω and an imaginary (inductive) part of approximately 144 Ω at 915 MHz, which means that the adaptation to the impedance of the chip becomes quite simple by using inductors near the power port. This is visible in Figure 5, where the adaptation circuit is shown in Figure 5c (consisting of two inductors: one series of 40 nH and one parallel of 109 nH), and in Figure 5d is shown the integration of the adaptation network between the antenna and the chip. The reflection loss (module of parameter S11) becomes very good at the working frequency, with an optimal power transfer between the logarithmic spiral and the RFID chip.

**Figure 6:**
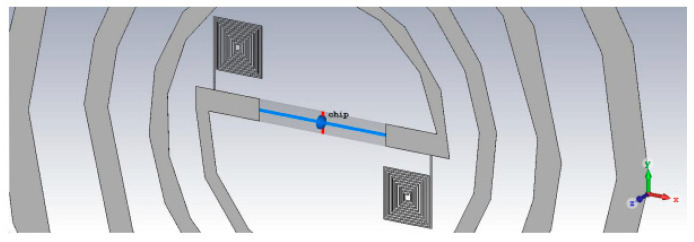
The electromagnetic design of the spiral antenna integrated with an RFID chip impedance adaptation network.

Figure 6 shows the design of the antenna with a symmetrical adaptation network, which is made up of two spiral inductors, the RFID chip being placed at their inner ends.

**Figure 7:**
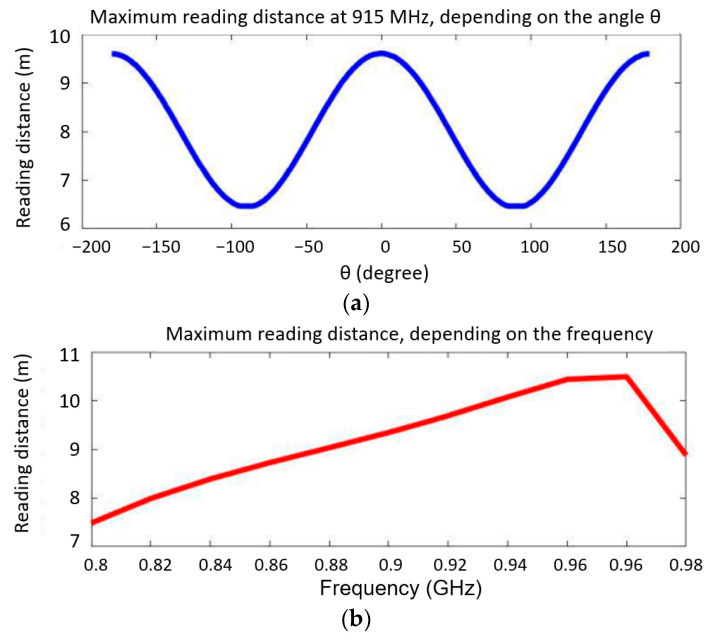
The maximum reading distance (**a**) at 915 MHz depending on the angle θ and (**b**) depending on the frequency.

Based on this design, the maximum reading distance of the entire RFID tag was calculated in the most unfavorable situation in terms of losses of the PET layer (Table 1, tan δ = 0.002). The maximum reading distance can be calculated as follows: by using Friis’ formula, assuming (in a realistic way) to have an optimal adaptation: (1) of the polarization between the antenna placed in the reader and the antenna placed in the label, and (2) of the impedance of the antenna in the tag with the RFID chip; the maximum reading distance *d_max_* is given by the Equation (2) [22]:

(2) $$d_{max}=\frac{\lambda }{4\pi }\sqrt{\frac{EIRP∙G_{e}}{P_{e}}}\;$$

where *λ* is the wavelength in free space, EIRP is the power apparently isotropically radiated by the antenna placed in the reader (in Europe, *EIRP* = 35 dBm or 3.2 W), *G_e_* is the gain of the designed spiral antenna placed in the tag, and *P_e_* is the power received by the spiral antenna. This latter power corresponds to the “READ sensitivity” value of −299 dBm specified in the NXP UCODE 8/8 mm chip manufacturer’s sheet. The values for *d_max_* depending on the angle θ (at 915 MHz) and depending on the frequency are shown in Figure 7. One can see that at 915 MHz (blue curve, Figure 7a), for an angle of 100° (between −50° and 50°) *d_max_* is at least 8 m, while in a perpendicular plane (θ = 0°) *d_max_* is over 9.5 m. In any case at an angle of 180° (between −0° and 90°) *d_max_* is at least 6.5 m.

Figure 7b (the red curve) shows that d_max_ is between 8.5 and 10.58 m in the 860–960 MHz range, which already represents a promising result for the design target.

### 2.2. Manufacturing of RFID Elements

**Figure 8:**
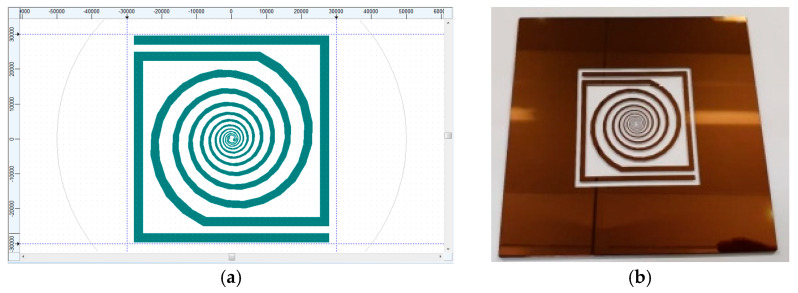
(**a**) Layout and (**b**) photolithographic mask of the RFID elements (antenna and symmetric attenuation network).

The fabrication of the antenna is performed using standard photolithography. The DWL 66fs Laser Lithography System (Heidelberg Instruments Gmbh Mikrotechnik, Heidelberg, Germany) was employed to obtain the photolithographic mask (Figure 8b) used to pattern the RFID elements (antenna and symmetrical attenuation network). Figure 8 shows the designed layout of the RFID elements (antenna with symmetrical attenuation network) and the fabricated photolithographic mask.

A Kapton^®^ HN foil (DuPont Electronics & Industrial, Newark, DE, USA) with a thickness of 25 µm and a size of 10 × 10 cm was used as a substrate for the RFID antenna. Although in the initial stages PET was proposed as substrate, during the manufacturing process it was found that the Kapton HN foil offers superior adhesion properties compared to the classical substrate (PET), thus allowing the realization of a metallic antenna with minimum fabrication defects. This foil was stretched perfectly on a glass substrate and glued to the edges with adhesive Kapton tape (used due to its good resistance to high temperatures). A thin layer of photoresist ma-N-1420 (negative resist, Microresist, Berlin, Germany) was deposited on the Kapton NH foil by centrifugation at 3000 rpm for 60 s, resulting in a thickness of the photoresist layer of 2 µm.

The exposure of the photolithographic mask for the RFID antenna was carried out with the Mask Aligner—MA6/BA6 (Suss MicroTec, München, Germany), using an exposure at the wavelength of 365 nm for 43 s. After exposure, the photoresist was developed in a special solution ma-D 533/S (Microresist, Berlin, Germany) for 70 s.

A layer of titanium/silver (Ti/Ag) with a thickness of 10/1000 nm was deposited on the surface of the patterned photoresist by the DC Sputtering method using the equipment Electron Beam Evaporation an DC sputtering system—AUTO 500 (BOC Edwards, Crawley, UK). The chamber pressure of the deposition installation was 1.82 × 10^−3^ Pa using an argon flow rate of 2.5 sccm at a power of 85 W. The Ti layer was used as an adhesion layer on the Kapton NH surface.

After obtaining the metal layer, the support with the Kapton NH foil was immersed in acetone for lift-off process, the result being the RFID antenna on a thin Kapton substrate. Finally, the Kapton adhesive tape used to fix the stretched foil on the glass substrate was removed.

**Figure 9:**
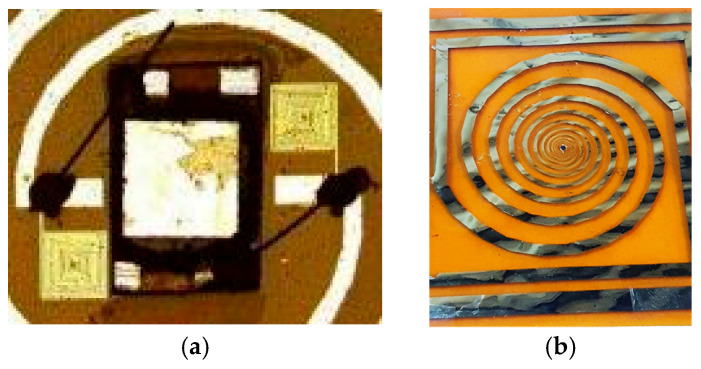
(**a**) Optical image after gluing gold wires with epoxy conductive paste; (**b**) final foil of the experimental RFID model.

For chip and wire soldering, a three-step process has been developed to achieve the assembly of chips and external components on foil. First, a conductive resin, H20E (Epoxy Technology Inc., Billerica, MA, USA), was used to make the interconnections between the integrated circuits and the silver plates. A double layer of 50 μm-thick dry adhesive, AR Clear 8932 (Adhesives Research, Inc., Glen Rock, PA, USA), was placed on the underside of the chip to fix it to the substrate. Next, the resulting structure was annealed in an oven at 120 °C for 20 min to harden the conductive resin. Moreover, the adhesion of the dry film improves with the temperature, so that the thermal treatment also served to better fix the chip on the substrate. In Figure 9, the RFID antenna and chip glued together with epoxy conductive paste (Figure 9a) and the final foil of the resulted experimental RFID model (Figure 9b) are shown.

## 3. Realization of the Multilayer Holographic Label

The manufacturing technology of a multilayer holographic smart label combines the processes for: (i) the holographic background, (ii) the nano-text type security elements, and (iii) the holographic security elements and the technology of passive RFID elements.

### 3.1. Anti-Counterfeiting Holographic Label

The technologies for obtaining a holographic background and high security elements of micro and nanotext used for security labels are described in [15,16]. Here we adapted those technologies by changing the standard substrate for DOE (metallized foil) with a semi-transparent reflective foil containing a zinc sulfide thin film. This foil is special for holographic security labels with a total or controlled destruction effect.

**Figure 10:**
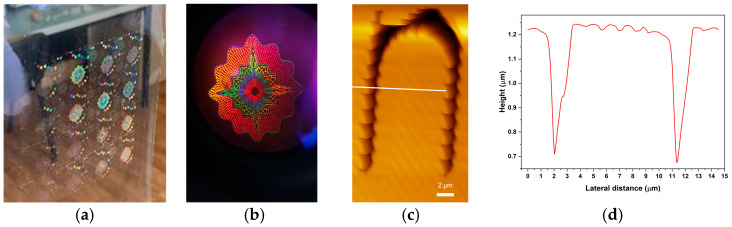
Developed holographic foil image: (**a**) overall image; (**b**) detail through the objective of the optical microscope; (**c**) AFM image of text with sub-micron line width “hidden” in the holographic background; and (**d**) profile.

Figure 10 shows the developed holographic background obtained on transparent holographic foil (Figure 10a) with a detail of a diffractive optical element (Figure 10b) and AFM image of text with sub-micron line width “hidden” in the holographic background (Figure 10c) with profile (Figure 10d).

### 3.2. Integration of the Classic Anti-Counterfeiting Label with the Developed RFID Elements

**Figure 11:**
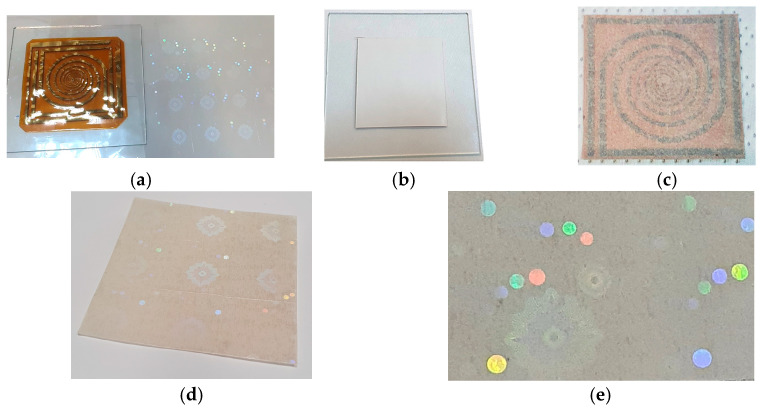
Integration stage: (**a**) antenna configured on kapton substrate with passive RFID chip and holographic foil; (**b**) layer attachment to increase the visual effect of the holographic elements; (**c**) intelligent multilayer hologram backside with adhesive and protective silicone paper; (**d**) multilayer intelligent hologram frontside; and (**e**) detail.

To obtain the proposed multilayer holographic label, the security components, i.e., the foil with holographic background and security elements of micro/nanotext, and the foil with the RFID elements were integrated. The integration of these elements was achieved by lamination. Ritrama adhesive was applied to the holographic foil and the RFID antenna. They were laminated together to obtain the structure presented in Figure 1. The integration of the components was performed taking care not to damage the holographic image or deform the antenna. A 20 µm-thick paper was inserted between the two foils to visually amplify the holographic effect and to hide the antenna. Figure 11 shows the integration stages of the security components: (a) antenna configured on Kapton HN substrate with passive RFID chip and holographic foil; (b) thin paper layer attachment to increase the visual effect of the holographic elements; (c) intelligent multilayer hologram backside with adhesive and protective silicone paper; (d) multilayer intelligent hologram frontside; and (e) detail.

Table 2 shows the types and thicknesses of the layers that compose the smart holographic label obtained in this work.

The results were validated by performing tests to determine the maximum identification distance using a commercial RFID reader (model ZT-STU-8280C produced by Scivos). This is a medium-distance UHF RFID proximity reader in the 865–928 MHz frequency band, mainly used for access control centres. The emission power of the reader is 1 W, adjustable by a dedicated software program. It is compatible with chips that support ISO 18000-6B and ISO 18000-6C (EPC Gen2) passive (865 MHz) IDC-1001UHF-GEN2 protocols.

The reading distance recommended by the manufacturer is approximately 5 m. In our tests, for the validation of the multilayer smart holographic labels obtained, this reader was optimized to have the ability to read at distances of up to 10 m. The reader can work in two modes: permanent reading that allows products to be monitored in real time, and triggered reading using a specific application to check the products at a certain time. Moreover, the reader has the possibility to be adjusted to the frequency and power of the emission. The measurements were performed using a face-to-face orientation at a 90° ± 20° angle in ambient temperature with normal atmospheric and surrounding electromagnetic fields conditions. The reading antenna was fixed on a vertical support in air, and the multilayer tag was attached with adhesive on a cardboard package of a product. The reading range was verified by varying the distance of the secured product with respect to the fixed UHF RFID reader antenna. The reading time of the label did not exceed 10 ms every 8 bytes, and the communication with the control centre was carried out through the RS232/RS 485/Wiegand 26/Wiegand 34 ports.

The tests to determine the maximum reading distance of the holographic labels were carried out both in a corridor of a production hall and inside of a mechanical workshop, to be able to evaluate the possible disturbances during the reading of the label, due to the obstacles present in the room. The obtained results for the reading distance were in the range of 9.2–10.2 m, exceeding the initial proposed objective of reaching a distance of at least 6 m.

## 4. Conclusions

In this work, an RFID element was designed, fabricated and integrated with a holographic label in order to create a smart tag with a high security level for product anti-counterfeiting protection. The smart label allows the reading of product information from a long distance (up to 10 m), thus assuring product surveillance during the supply chain. The multilayer tag was obtained in three steps: (1) realization of the holographic labels with different security elements (such as holographic background, foreground, and nanotext) on a semi-transparent foil with zinc sulfide thin film; (2) fabrication of the RFID antenna on the polymer foil and integration with chip; and (3) integration of the two developed components.

The classical holographic technology which uses a metallized foil as substrate for DOE was adapted and optimized to obtain the same high-quality holographic elements on a semi-transparent reflective foil with a total or controlled destruction effect. This foil was selected to maximize the RFID antenna footprint and to facilitate the transmission of the electromagnetic signal through the label, thus achieving up to a 10 m reading distance, with a 6 cm × 6 cm label. The developed RFID antenna consists of a passive tag specifically designed to operate in the 860–960 MHz frequency range with a bending possibility of no more than 90° integrated patterned on a Kapton NH foil. To store the product information on the smart label, a commercial RFID chip (with a typical impedance at 915 MHz of ZC = 19 − 234 j Ω) was integrated with the fabricated antenna. The RFID elements were integrated with the semi-transparent foil containing the holographic security structures by lamination using a Ritrama adhesive. In addition, a paper layer was inserted between the two foils to increase the holographic effect and hide the antenna.

After integration, a smart multilayer label was obtained. This tag is extremely difficult to forge and allows for quick and remote product surveillance and authentication.

The next step in our study is to develop and integrate a temperature sensor with the RFID elements to ensure proper storage for temperature-sensitive products.

## 5. Patents

The following patent application has resulted from the work reported in this manuscript: Brandus Comanescu, Mihaela Pelteacu, Dana Cristea, Catalin Parvulescu, Roxana Tomescu, Process of integrating long-range RFID elements into multi-layer smart holographic labels to increase the degree of security, A/00264 from 13.05.2022, State Office for Inventions and Trademarks, Bucharest, Romania.

## Figures and Tables

**Table 1:** Electrical characteristics of the materials for the holographic label.

Material	Permitivity (ε)	The Tangent of the Loss Angle (tan δ)
Adhesive	4	0.02
ZnS	8.9	0.007
PET	3.5	1.4 × 10^−6^–0.002

**Table 2:** Component thicknesses of the multi-layer smart holographic label.

Component	Thickness [µm]
Holographic label in semi-transparent foil	35
Adhesive	15
Film to increase the holographic effect and hide the antenna	20
Adhesive	15
RFID label consisting of Antenna and RFID Chip	50
Adhesive for attaching on the product	15

## Data Availability

Not applicable.
